# Efficacy of prepartum vaccination against neonatal calf diarrhea in Nelore dams as a prevention measure

**DOI:** 10.1186/s12917-022-03391-5

**Published:** 2022-08-22

**Authors:** Filipe Aguera Pinheiro, Nathália Decaris, Viviana Parreño, Paulo Eduardo Brandão, Henderson Ayres, Viviani Gomes

**Affiliations:** 1 Department of Internal Medicine, College of Veterinary Medicine and Animal Science, University of São Paulo, 87, Prof. Dr. Orlando Marques de Paiva Avenue, Cidade Universitária, Butantã, Sao Paulo, 05508-270 Brazil; 2 INCUINTA. Instituto de Virologia e Tecnológicas, IVIT. CICV y A. Instituto Nacional de Tecnología Agropecuaria (INTA). Unidad ejecutora INTA-CONICET, Nicolas Repetto y de los Reseros s/n, Postal code 1686 Buenos Aires, Argentina; 3 Department of Preventive Veterinary Medicine and Animal Health of Internal Medicine, College of Veterinary Medicine and Animal Science, University of São Paulo, 87, Prof. Dr. Orlando Marques de Paiva Avenue, Cidade Universitária, Butantã, São Paulo, 05508-270 Brazil; 4 MSD Animal Health, 296, Dr. Chucri Zaidan Ave, Vila Cordeiro, São Paulo, São Paulo 50030-000 Brazil

**Keywords:** Bovine rotavirus, Bovine coronavirus, Calves, Serological response

## Abstract

**Background:**

Neonatal calf diarrhea (NCD) is the leading cause of calf morbidity and mortality in beef cattle. Cow’s vaccination in last stage of pregnancy is one of the most important measures to mitigate the risk of NCD outbreaks. The aim of this study was to evaluate the efficacy of prepartum single dose vaccination against NCD, especially Bovine Rotavirus type A (BoRVA) and Bovine Coronavirus (BCoV), in Nelore dams and offspring. A total of 117 pregnant cows (*n* = 81) and heifers (*n* = 36) were distributed in two groups, vaccinated (VAC: cows = 40; heifers = 19) and non-vaccinated (NVAC: cows = 41; heifers = 17). Vaccination occurred between 60 to 50 days before the expected calving date with a single dose of a water-in-oil (W/O) vaccine, and NVAC group received a dose of saline solution 0.9%. Blood samples were collected before vaccination and 30 days after to evaluate the antibody (Ab) response. Specific IgG1 Abs against BoRVA and BCoV were measured by using an Enzyme Linked Immuno Sorbent Assay (ELISA). Calves’ births were monitored, and the transference of passive immunity was evaluated. Diarrhea was monitored in the first 30 days of age, and fecal samples were collected for identification of the etiological agent.

**Results:**

Higher titers of IgG1 Ab against BoRVA and BCoV was observed in the VAC group than NVAC group in the cow (*P* < 0.0001) and total dams categories (*P* < 0.0001). The titer of specific IgG1 Abs in the calves’ serum reflected the dams response, observing higher IgG1 Ab titers for BoRVA (*P* < 0.0016) and BCoV (*P* < 0.0095) in the offspring born to VAC cows and higher IgG1 Ab titers for BoRVA(*P* < 0.0171) and BCoV (*P* < 0.0200) in the offspring born to VAC total dams. The general incidence of diarrhea observed was 18.6% (11/59) and 29.3% (17/58) in the calves born to the VAC and NVAC group, respectively.

**Conclusions:**

Prepartum vaccination with a single dose of the vaccine tested increased the titers of IgG1 Ab against BCoV and BoRVA, and it could be used as a preventive strategy to decrease the NCD occurrence in Nelore calves.

**Supplementary Information:**

The online version contains supplementary material available at 10.1186/s12917-022-03391-5.

## Background

Neonatal calf diarrhea (NCD) is one of the most important diseases in both dairy and beef cattle, which is a multifactorial disease and brings high economic losses, not only from mortality, but also for the cost of medication to treat sick calves and impacts on production [1]. In beef calves, diarrhea is more frequent in herds that use artificial insemination (AI) and have seasonal calving due to the concentration of births in a small period of the year, which facilitates the transmission of pathogens [2]. Failure of passive immunity transfer (FPIT) is a predisposing factor, and is more frequent in calves born from heifers, as they often have less maternal ability or less volume of colostrum presenting low immunological quality [3, 4]. The most important pathogens involved in NCD are enterotoxigenic *Escherichia coli* (ETEC K99), *Cryptosporidium parvum*, Bovine Rotavirus type A (BoRVA) and Bovine Coronavirus (BCoV) [5]. In Brazilian beef calves, BoRVA has been the most predominant pathogen identified in diarrhea outbreaks, which demonstrate the importance of this agent in the occurrence of NCD [2, 6].

The basic pillars for the prevention of NCD are the increase of the host’s immunity and reduce the load of infectious agents in the environment [1]. Prepartum vaccination of dams is one of the main strategies to improve the host’s immunity. The objective is to increase the concentration of specific immunoglobulins in colostrum and provide passive immune transfer of immunoglobulin to agammaglobulemic neonates [7]. However, the effectiveness of prepartum vaccination to prevent NCD is very controversial, with studies that show good results and others that are not so favourable. In one study carried out using a vaccine containing live BoRVA and BCoV, and *E. coli* F5 bacterin, there was no differences in the occurrence of NCD and mortality rates in vaccinated and unvaccinated calves on dairy farms. Furthermore, colostral antibodies to BoRVA and BCoV were similar in both groups [8]. Even so, several other studies have reported that pregnant cows vaccinated against NCD, specifically BoRVA and BCoV had increased titers of antibodies in colostrum and offspring [9–11].

In beef cattle, one field study evaluated the concentration and persistence of antibodies in colostrum and milk against BoRVA, BCoV and *E. coli* F5 in two groups (vaccinated and no vaccinated animals). In this study, was observed higher titers of specific antibodies against these agents in the serum and colostrum from cows and in the serum of calves in the vaccinated group. Despite the higher titers in the vaccinated group, this research did not evaluate the effectiveness of the vaccine formulations to prevent the occurrence of NCD [7]. Another research evaluated the effectiveness of a commercial vaccine containing the G6P [5] genotype of BoRVA in dairy cattle and found efficacy in prepartum vaccination to increase specific antibodies against these agents in the colostrum of cows and in the serum of calves in the group of vaccinated animals. However, colostral immunity was not sufficient to prevent NCD, with a higher occurrence in the vaccinated group [12].

Another problem that has been demonstrated in recent studies is the difference between strains used in the production of vaccines and strains found in the field, which puts in doubt the effectiveness of vaccination [13]. One study evaluated the occurrence of NCD by BoRVA in a herd regularly vaccinated with a commercial vaccine containing the BoRVA G6P [5] genotype and found 62.3% of the animals (76/122) positive for the G10P [11] genotype [14]. Another verified the occurrence of BoRVA diarrhea in a Turkish herd also regularly vaccinated against strain G6P [5] with high morbidity and mortality rates associated with the infection by strain G8P [5, 15].

In this context, the objective of this research was to evaluate the effectiveness of the prepartum vaccination with a commercial vaccine composed of inactivated BoRVA, inactivated BCoV and ETEC K99 to protect calves against NCD. Our targets were to evaluate specific IgG1 antibody responses against BoRVA and BCoV in the dams and the transference of this passive maternal antibodies to their offspring. In addition, as a secondary objective, the etiological diagnosis of the main etiologic agents causing NCD in the studied herd was carried out. The hypothesis was that prepartum vaccination optimizes the transfer of passive colostral immunity and help to prevent diarrhea occurrence in Nelore calves. To the best of our knowledge, studies regarding the effectiveness of prepartum vaccination in beef cattle for prevention of NCD are scarce; furthermore, this research is the first in Nelore’s breed.

## Results

### Dams

#### General characterization and adverse reaction from vaccination

The median interval between vaccination and parturition presented by heifers, cows and total dams from this research were 54 days (45–93 days), 64 days (46–111 days) and 61 days (45–111 days), respectively. All dams (heifers and cows) from this research calved by eutocic parturation. Thirty days after vaccination, all pregnant heifers and cows were observed for the second time during the blood sampling. It was possible to detect some local reaction in the point of vaccine injection in 31.57% (6/19) of heifers. On the other hand, none of the multiparous cows had similar adverse effect after vaccination.

#### Serological response from dams

The effect of groups, times and interaction between groups and times on IgG1 Ab against BoRVA and BCoV from the vaccine antigens are shown in the Table 1. For BCoV, it was possible to detect effect of groups (*P* < 0.0144) and interaction between groups and times (*P* < 0.0001) in the cows, and effect of groups (*P* < 0.0389) and interaction between groups and times (*P* < 0.0001) in the total dams categories. For specific antibodies against BoRVA, it was possible to detect effect of time (*P* < 0.0220) and interaction between groups and times (*P* < 0.0066) in the heifers, effect of time (*P* < 0.0001) and interaction between groups and times (*P* < 0.0001) in the cows, and effect of time (*P* < 0.0001) and interaction between groups and times (*P* < 0.0001) in the total dams categories.

**Table 1:** Effect of groups, times and interaction (groups*times) on the titers of specific IgG1 antibodies against BCoV and BoRVA (Log10) in Nelore dams

Agents	Dams Categories	Times	N Obs	Mean	Std Error	Groups	Times	Groups*Times
BCoV	Heifers	60PP	36	4.03	0.09	0.6383	0.0220	0.0066
		30PP	36	4.21	0.08			
	Cows	60PP	81	4.46	0.05	0.0144	0.8315	< 0.0001
		30PP	81	4.47	0.06			
	Total Dams	60PP	117	4.47	0.04	0.0389	0.4581	< 0.0001
		30PP	117	4.51	0.05			
BoRVA	Heifers	60PP	36	4.03	0.09	0.6383	0.0220	0.0066
		30PP	36	4.21	0.08			
	Cows	60PP	81	4.31	0.05	0.1400	< 0.0001	< 0.0001
		30PP	81	4.46	0.05			
	Total Dams	60PP	117	4.22	0.04	0.4794	< 0.0001	< 0.0001
		30PP	117	4.38	0.04			

Student T Test; 60PP – 60 days before expected calving; 30PP – 30 days before expected calving

**Fig. 1:**
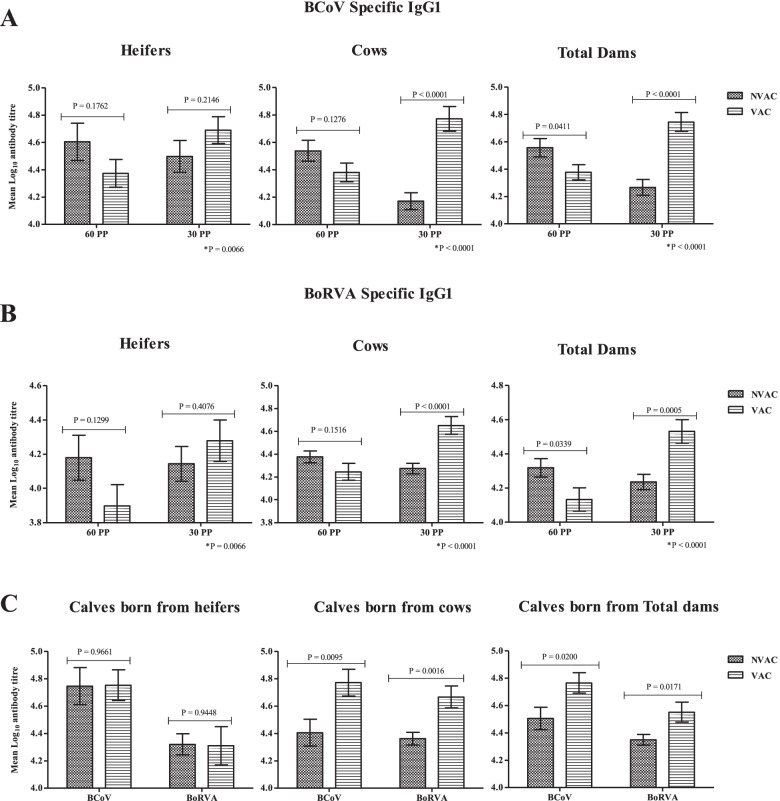
Mean (±STD Err) of IgG1 Ab against BCoV and BoRVA (Log10) in Nelore dams and offspring from NVAC and VAC groups. Legend: The difference between groups in each time of evaluation was detected by using T Student test; 60PP – 60 days before expected calving; 30PP – 30 days before expected calving; *P - interaction between groups and times (Groups x Times) by using PROC MIXED; all analyzes were considered significant when *P* < 0.05

The results of the IgG1 Ab titration against BCoV and BoRVA at the time of vaccination (60 days prepartum) and after vaccination (30 days prepartum) are shown in Fig. 1 A and B. At the time of vaccination, the basal mean IgG1 Ab titers to BCoV and BoRVA were higher in the NVAC vs. the VAC group, for both categories, heifer and cows, but not significantly so (*P* > 0.05). However, when pooling the data, total dams Ab titers for both viruses in NVAC group was significantly higher than in the VAC group (BCoV, *P* = 0.0411 and BoRVA, *P* = 0.039). After vaccination, the IgG1 Ab titers against BCoV and BoRVA were higher in the VAC than NVAC group in both categories, being significantly higher for the vaccinated than in non-vaccinated cows and total dams. In the heifer’s category, even when the Ab titer between NVAC and VAC after vaccination was statistically similar, in the VAC group there was a significant increase in the Ab titer from 60PP to 30PP (*P* < 0.05), while IgG1 Ab titers decreased or remained similar in the non-vaccinated control group.

### Calves

#### General description

All calves included in this study (*n* = 117) were born without any calving assistance in both heifer (*n* = 36) and cows (*n* = 81) categories. Within of this total, male represented 65 calves and 52 are female. The body weight at D2-D3 was similar between NVAC (34.88 ± 0.58) and VAC (33.64 ± 0.51) groups in all dam’s categories (*P* > 0.05). In the clinical evaluation performed on D2-D3, all calves presented vital parameters within normal range, hydrated and with normal fecal consistency. On the other hand, 7.69% (9/117) calves had umbilical inflammation.

#### Total serum protein (TSP)

The concentration of TSP Means (± STD Error) after colostrum feeding is shown in Table 3. TSP concentration (g/dL) was similar between experimental groups NVAC and VAC in heifer (*P* = 0.63), cows (*P* = 0.62) and total dams (*P* = 0.89), according to the Student T test. The failure of transfer of passive immunity (FPIT) rate was also calculated using different cut-off for TSP, however the result found was similar between experimental groups in all maternal categories (Table 2).

**Table 2:** Concentration of total serum protein g/dL (Mean ± STD Err) and rate % (n/total) of failure of transfer of passive immunity (FTPI) in Nelore calves born from VAC and NVAC dams in the pre-partum period, according to the different cut-off for total serum protein (g/dL)

Dams Category	Groups	STP	Protein Cut-Point Total Protein (g/dL)			
			5.5	6.0	6.5	7.0
Heifers	NVAC	6.8471 ± 0.2111	11.76 (2/17)	23.53 (4/17)	29.41 (5/17)	47.05 (8/17)
	VAC	7.0000 ± 0.2284	0.00 (0/19)	15.79 (3/19)	36.84 (7/19)	52.63(10/19)
	*P*-value	0.6287	0.216*	0.684*	0.637	0.738
Cows	NVAC	7.0341 ± 0.1429	4.87 (2/41)	14.63 (6/41)	31.70 (13/41)	39.02 (16/41)
	VAC	6.9350 ± 0.1402	7.50 (3/40)	7.50 (3/40)	35.00 (14/40)	52.5 (21/40)
	P-value	0.6220	0.675*	0.482	0.753	0.320
Total	NVAC	6.9793 ± 0.1180	6.89 (4/58)	17.24 (10/58)	31.03 (18/58)	41.38 (24/58)
	VAC	6.9559 ± 0.1191	5.08 (3/59)	10.16 (6/59)	35.59 (21/59)	52.54 (31/59)
	P-value	0.8894	0.717*	0.266	0.601	0.307

Sig. = *P* value Fisher’s Exact Test*/Chi-Square Test

#### IgG1 Ab titers against BoRVA and BCoV

The IgG1 Ab titers against BoRVA and BCoV are shown in Fig. 1C. In the calves born from heifers, the IgG1 Ab titer were similar between NVAC and VAC groups (BCoV, *P* = 0.961, BoRVA *P* = 0.9448). On the other hand, calves born from of cows from VAC group had significantly higher IgG1 Ab titers against BCoV and BoRVA than NVAC group (BCoV, *P* = 0.0095, BoRVA *P* = 0.0016), and calves born to total dams from VAC group had significantly higher IgG1 Ab titers against BCoV and BoRVA than NVAC group (BCoV, *P* = 0.0200, BoRVA *P* = 0.0171).

#### Diarrhea incidence during the neonatal period

The general occurrence of diarrhea in the calves born to total dams in the VAC and NVAC groups was 18.6% (11/59) and 29.3% (17/58) respectively (*P* = 0.18). In the calves born from heifers, diarrhea incidence was 26.30% (5/19) and 41.20% (7/17) in the VAC and NVAC groups, respectively (*P* = 0.34). Calves born from cows had 15% (6/40) and 24.40% (10/41) in the VAC and NVAC groups, respectively (*P* = 0.29). Despite of the higher frequencies of diarrhea in calves born to NVAC groups, there was no statistical difference in any of the categories (*P* > 0.05).

#### Etiological diagnosis of diarrhea

A total of 56 fecal samples was processed for the detection of BoRVA, BCoV, *E. coli* K99 and Cryptosporidium spp. (Table 3). From these, 28 fecal sample was from diarrheic calves and 28 was from health calf presenting similar age. The samples from health calves were collected for comparation with diarrheic calves. In the diarrheic calves, BoRVA was the main challenge detected in our experiment. In heifers’ category, it was detected 60% (3/5) and 85.71% (6/7) of positive samples for BoRVA, respectively, in NVAC and VAC groups. In cows’ category, it was detected 66.66% (4/6) and 60.00% (6/10) of positive samples for BoRVA, respectively, in VAC and NVAC groups. Within total dam’s category, it was detected 63.63% (7/11) and 70.58% (12/17) of positive samples for BoRVA, respectively, in VAC and NVAC groups. BCoV was the second more important challenge in the Nelore herd investigated during this research. This agent was only detected in the cows’ category, observing 16.66% (1/6) and 30.00% (3/10) of positive samples in the VAC and NVAC groups, respectively. A mixed infection between BoRVA and BCoV 9.09% (1/11) was identified in the NVAC group in the cow category. *Cryptosporidium* spp. and *Escherichia coli* K99 were not detected in diarrheic feces from any calves born from VAC and NVAC dams, according to the methodology adopted in this research.

**Table 3:** Etiological agents in Nelore calves born from non-vaccinated and vaccinated dams in the pre-partum period

Dams Categories	Pathogens	Calves manifesting diarrhea		Healthy calves
		VAC	NVAC	
Heifers	BoRVA	60.00 (3/5)^a^	85.71 (6/7)^a^	16.66 (2/12)^b^
	BCoV	0.00 (0/5)	0.00 (0/7)	0.00 (0/12)
	*Cryptosporidium* spp.	0.00 (0/5)	0.00 (0/7)	8.33 (1/12)
	*E. coli* K99	0.00 (0/5)	0.00 (0/7)	0.00 (0/12)
	*Eimeria* spp.	20.00 (1/5)	0.00 (0/7)	8.33 (1/12)
	BoRVA/BCoV	0.00 (0/5)	0.00 (0/7)	0.00 (0/12)
Cows	BoRVA	66.66 (4/6)	60.00 (6/10)	31.25 (5/16)
	BCoV	16.66 (1/6)	30.00 (3/10)	18.75 (3/16)
	*Cryptosporidium* spp.	0.00 (0/6)	0.00 (0/10)	6.25 (1/16)
	*E. coli* K99	0.00 (0/6)	0.00 (0/10)	0.00 (0/16)
	*Eimeria* spp.	0.00 (0/6)	0.00 (0/10)	0.00 (0/16)
	BoRVA/BCoV	0.00 (0/6)	10.00 (1/10)	6.25 (1/16)
Total dams	BoRVA	63.63 (7/11)^a^	70.58 (12/17)^a^	25.00 (7/28)^b^
	BCoV	9.09 (1/11)	17.64 (3/17)	10.71 (3/28)
	*Cryptosporidium* spp.	0.00 (0/11)	0.00 (0/17)	7.14 (2/28)
	*E. coli* K99	0.00 (0/11)	0.00 (0/17)	0.00 (0/28)
	*Eimeria* spp.	0.00 (0/11)	9.09 (1/11)	3.57 (1/28)
	BoRVA/BCoV	0.00 (0/11)	9.09 (1/11)	3.57 (1/28)

Different letters on the same line indicate statistical difference between groups (Sig. = *P* < 0.05 Fisher’s Exact Test*/Chi-Square Test)

The same etiological agents also were investigated in the fecal samples from healthy calves. Within the total samples evaluated (*n* = 28), it was possible to detect 25% (7/28) and 10.71% (3/28) of positive results for BoRVA and BCoV, respectively. *Cryptosporidium* spp. was identified in 7.14% (2/28) samples in health calves, despite of this agent was not detected in the diarrheic fecal samples. The statistical analysis between experimental groups for diarrhea pathogens showed difference only for BoRVA in the heifers and total dams’ category, with higher incidence in the diarrheic calves vaccinated or nor comparing with healthy control group (*P* < 0.05). The analysis of the BoRVA VP7 gene performed on samples from three positive animals identified the presence of the G6 genotype, with 92.3% nucleotide identity between the vaccine and the field strain.

## Discussion

The reproductive protocol using fixed-time artificial insemination ensured the window time between vaccination and parturation recommended by the label of the commercial product.

The vaccination protocol followed the manufacturer instructions applying only a single dose of vaccine. The vaccine has aluminium hydroxide and MontanideTM ISA70 VG as adjuvant. The MontanideTM ISA series of adjuvants include the water-in-oil (W/O) emulsions, MontanideTM ISA 70 VG (ISA 70) and MontanideTM ISA 71 VG (ISA 71). Both formulations are mineral oil-based solutions incorporating a highly refined mannitol/oleic acid emulsifier, which have been successfully applied to enhance immune response of vaccines against pathogens of poultry, cattle, and small ruminants [16].

Despite the occurrence of a local vaccine reaction in some animals, this finding is not of great importance. Vaccine reactions are consequences mainly of the type of vaccine, adjuvants used as vehicles, inoculation routes, type of needle and contamination [17]. However, in our study, a single syringe and needle was used for each animal, in addition to antisepsis at the application site, which reduces the occurrence of contamination. In addition, MontanideTM ISA adjuvant series has been associated with low local reaction [16].

The cow’s vaccination with a single dose of vaccine against NCD induced an optimal humoral immune response against BoRVA and BCoV in cows and total dams’ categories. These results agree with several other papers which demonstrate that the prepartum vaccination of cows with formulation containing diarrhea pathogens induces an increase on the levels of specific antibodies in the serum and colostrum of vaccinated dairy and beef cows [7, 18]. The specific IgG1 Ab responses in heifers was also very good when analysing the increment between pre and post vaccination in the vaccinated group. However, the higher basal titer in the non-vaccinated group and well as the small number of heifers included in this experiment explain the absence of statistical difference between VAC and NVAC in this specific animal category. These results disagree with the results from another study, which found an increase in the levels of specific antibodies against BoRVA in primiparous Holstein cows [12]. It is worth mentioning that the experimental design from these studies was different considering breed, diet and production system. In addition, Nelore heifers that are bred in an extensive manner are characterized by the difficult handling and his management may cause stress. Stress can lead to an increase in the serum concentration of cortisol and a decrease in the immune response, which can lead to an increase in the occurrence of vaccine failures [19]. A two doses schedule for heifers will warrant vaccine efficacy?

The calves’ TSP measurement was performed with the intention of evaluating the rate of occurrence of FPIT. In beef calves, the literature about FTPI and the risk factors for its occurrence is very scarce. Also, there is some differences between beef and dairy cattle in relation to colostrum quality and passive immunity transfer. The immunoglobulin concentration in beef cows remains relatively constant and independently of colostrum volume produced by the dams [20], however, in dairy cattle there is an inverse proportionality between the amount of colostrum produced and the concentration of immunoglobulin (that is, the higher the production, the lower the quality). The number of cow deliveries is another factor that affects both the quantity and the concentration of specific immunoglobulins in colostrum from beef cows. Primiparous animals had lower yields and concentrations of specific antibodies than multiparous cows [20]. Unfortunately, it was not possible to measure colostrum production or collect samples to determine the antibodies concentration due to the aggressive behaviour of Nelore breed, especially in the immediate post-partum moment.

The ideal cut-points for total protein (g/dL) to define FPIT are widely varied among the authors and varying in beef or dairy calves. However, there is a consensus that the minimum adequate total protein concentration is 5.2 g/dL, which can vary between 5.0 and 5.5 g/dL [21]. According to a meta-analysis in dairy calves, the minimum cut-off for total serum protein to obtain the minimum number of false negatives is 5.5 g/dL (instead of 5.2 g/dL) measured by using an optical refractometer to exclude FTIP [22]. In our study, the rate of FPIT by using 5.5 or 6.0 g/dL as cut-off was similar between VAC and NVAC groups in all categories of dams, according to the Student T test used to contrast the groups means. These results ensure that the IgG1 Ab titers against BCoV and BoRVA found in the calves’ serum were not influenced by FTPI. In the calves, the IgG1 Ab titers against BCoV e BoRVA were similar between NVAC and VAC groups in heifer category (*P* > 0.05) and higher in the VAC group in cows and total dams category (*P* < 0.05). Based on these results, it is possible to state that the response of calves after ingestion of colostrum corresponds to the titer of IgG1 Ab detected in serum of all categories (heifers, cows and total dams) after vaccination. These results corroborate with several other authors who found that specific antibodies against BoRVA and BCoV are transferred efficiently to the offspring through colostrum intake [7, 10, 12].

In Brazil, there are no studies evaluating the occurrence of neonatal diarrhea in beef calves from vaccinated and unvaccinated dams. In addition, studies evaluating the incidence of neonatal diarrhea in beef herds are scarce and outdated. In a longitudinal study carried out in beef calves, following the animals throughout the first 9 weeks of life, the highest occurrence of diarrhea was identified between the 6th and 7th week, with a peak occurrence in the 6th week (around 22%) [23]. These results are similar to the rate of diarrhea observed in our study for cows’ categories in the NVAC group (24%), however the proportion of fecal score 2 and 3 was high in NVAC heifer category (around 41%). The rate of diarrhea in VAC group was lower than NVAC calves, however the low number of animals included in our experiment did not allow to identify statistical differences between experimental groups for any cattle categories. According to the incidence of diarrhea observed, we calculated the ideal number of calves to find statistical difference in future projects by using two proportions test performed on the R-3.6.3 software for windows. The ideal number of calves was 314 (VAC = 157 / NVAC =157) in heifers’ category, 606 (VAC = 303 / NVAC = 303) in cows’ category, and 498 (VAC 249 / NVAC = 249) in total dams. These results are shown in supplementary material (Table 1).

In general, our results showed a good vaccine response in the total number of animals and in the multiparous category, with a tendency towards a lower occurrence of diarrhea in the animals of the VAC group. However, these results reflect only the herd studied. Brazil is a country of large extensions, with different types of cattle raising and environmental differences, therefore, the microbiological challenges and the incidence of diarrhea in different herds can vary greatly, requiring broader studies that include different herds to validate the effectiveness of vaccination to prevent diarrhea in different properties.

The herd selected to develop this experiment has an endemic profile for diarrhea pathogens, and we did not identify an outbreak profile through the field step of this research. On the other hand, one study has reported an outbreak of diarrhea in Nelore calves (*Bos indicus*) up to 30 days old, from the state of Mato Grosso do Sul, Brazil, observing high rates of morbidity (60%) and lethality (7%) [2]. Another study also reported an outbreak of neonatal diarrhea with high morbidity (80%) and mortality (12%) among 1100 beef calves up to 30 days of age [6].

In Brazil, the reproductive management with breeding season and use of FTAI (Fixed-time artificial insemination) protocols can be a risk for neonatal diarrhea, because these protocols promote a concentration of the birth in determinate season of the year, favouring the transmission of enteric pathogens [2]. The most of neonate from our experiment were born in the beginning of the calving season and it can justify the low frequency of diarrhea detected in our research.

The first isolation of the BoRVA from diarrheal feces in beef calves in Brazil reported frequencies of positive samples between 41.7–82.4% [24]. In a longitudinal study carried out on Nelore herds, BoRVA was detected in 11% (11/100) of the analysed fecal samples [23]. Another study had evaluated the presence of BoRVA in beef and dairy calves over 10 years (2006–2015), and these authors found a frequency of 27.4% of positive results [25]. In this same study, the frequency of BoRVA-positive fecal samples in diarrheic calves from beef herds (31.9%) was higher than in dairy herds (17.4%) [25]. Other studies conducted in Brazil have identified BoRVA as the causative agent of diarrhea in 80% of the samples and 53.3% of the samples [2, 6]. Therefore, our data corroborate the findings of other authors regarding the frequency of BoRVA as a causative agent of neonatal diarrhea, highlighting the importance of this agent in national beef herd.

BCoV is identified as an important cause of diarrhea in calves [26, 27]. In Brazil, several studies have identified BCoV as an important causative agent of neonatal diarrhea in both beef and dairy herds [28, 29]. The presence of BCoV was identified in 14% of the feces samples from Nelore calves in one study. In addition, this agent has been identified in association with other enteropathogens, mainly BoRVA [23]. Another study identified a frequency of 33.3% (31/93) of BCoV in calf feces aged 0 to 60 days old from different regions of Brazil [30]. These results agree with the findings of our study.

Few studies are available in the literature evaluating the importance of *Cryptosporidium* spp. as a causative agent of neonatal diarrhea in beef calves. In the Formiga city (Minas Gerais) *Cryptosporidium* spp. was detected in only 5.3% (16/300) positive calves by using molecular characterization [31]. Other studies reported a frequency between 17.1 and 21.62% [32–34]. The differences regarding the frequencies reported for *Cryptosporidium* spp. can be explained using different techniques to identify this agent. In addition, risk factors related to the management and environmental conditions can influence the occurrence of this pathogen in different herds. Milk feeding system for artificially rearing calves is also a risk factor for infection caused by *Cryptosporidium* spp.*,* since oocysts can remain viable for a long period [32, 33]. Early exposure of calves to water in the first days of life can directly affect infection rates [33]. In our study, neonate Nelore calves were raised in a natural system (huge pasture) and it could prevent the *Cryptosporidium* spp. infection.

The small number of positive diarrheic fecal samples for *Cryptosporidium* spp. shows that this agent has not a great importance as a diarrheic agent in the Nelore herd where this study was conducted. The technique used to detect *Cryptosporidium* spp. in our study has high specificity, but it would be interesting to process the fecal samples by using a more sensitive technique such as PCR.

Enterotoxigenic *E. coli* (ETEC) is described worldwide as a cause of neonatal diarrhea in cattle. This pathotype basically has two virulence factors (fimbriae) that facilitate its connection to the intestinal epithelium which are F5 (K99) and F41, in addition to the production of a thermostable toxin (Sta). Fimbria F5 (K99) have been considered the virulence factor most important from ETEC [35]. The detection of this fimbria was identified in 5.8% (4/69) of *E. coli* strains isolated from diarrheic feces in beef calves [23]. Another study also identified the K99 fimbria in 7.3% of *E. coli* strains from diarrheic fecal samples in beef calves [36]. There are other studies that have not identified the presence of fimbria K99 in *E. coli* isolates from diarrheic fecal samples in calves [37], which agree with our results, and suggests the involvement of other colonization factors such as K88, 987P, F17 and F40 for the development of Colibacillosis in neonatal calves [23]. The same etiological agents also were investigated in the fecal samples from healthy calves. Within the total samples evaluated (*n* = 28), it was possible to detect 25% (7/28) and 10.71% (3/28) of positive results for BoRVA and BCoV, respectively. *Cryptosporidium* spp. was identified in 7.14% (2/28) samples in health calves, despite of this agent was not detected in the diarrheic fecal samples. These findings corroborate with other study who found these same agents in healthy animals as well [23].

The results of the analysis of the BoRVA VP7 gene shows that the vaccine antigen typed as G6P [5] showed a 92.3% nucleotide identity with the field virus also classified as G6, showing that despite the existence of reports of the occurrence of other variants [14, 15], the vaccine contains the viral strain of greatest occurrence.

## Conclusion

The dam’s vaccination in the prepartum period increased the titers of IgG1 Ab against BCoV and BoRVA in this herd, and it could be used as a strategy to reduce the occurrence of the NCD in Nelore calves, since these are the most prevalent diarrhea-causing agents.

## Methods

### Ethical approval

This research was conducted with the ethical approval of The Animal Use Ethics Committee of the Faculty of Veterinary Medicine and Animal Science of the University of São Paulo (Approval Number 4962250618).

### Herd information and study design

**Fig. 2:**
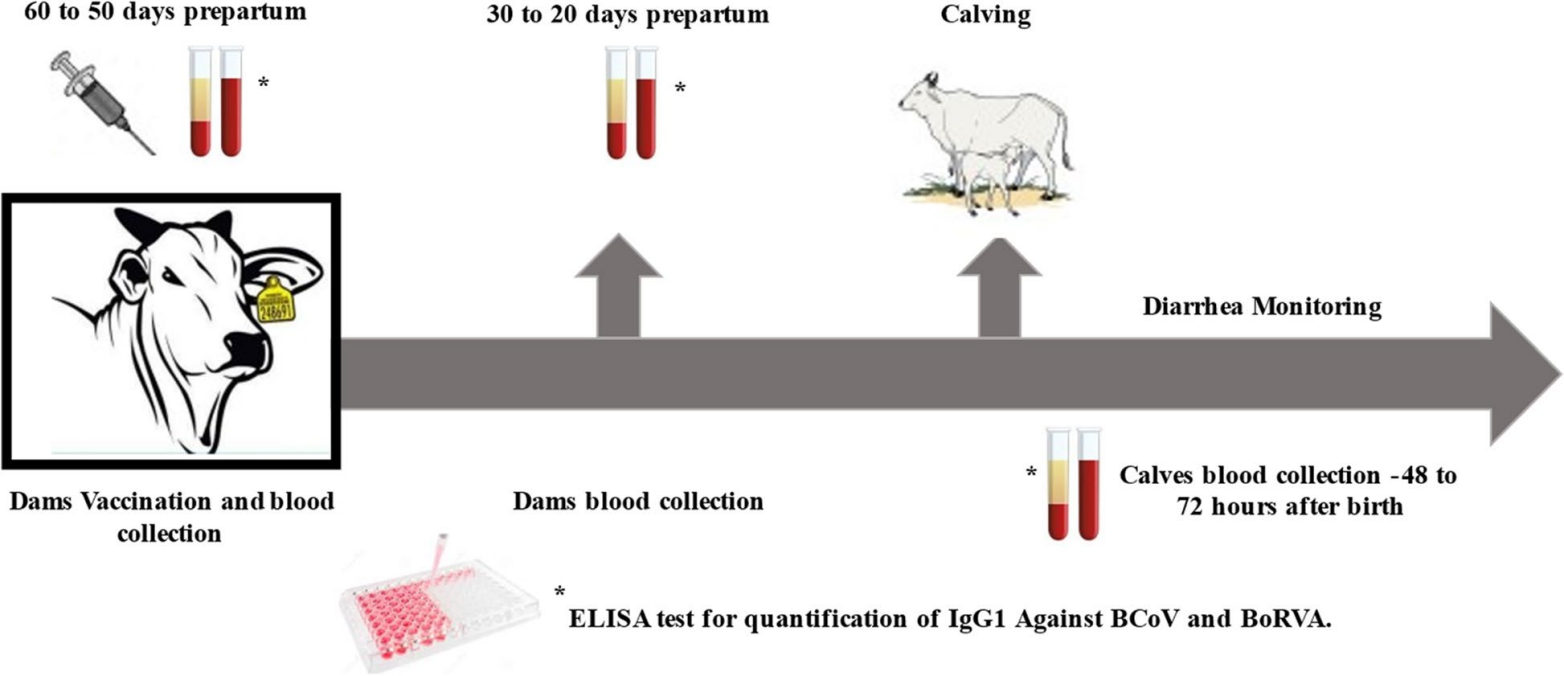
Study design, from vaccination to diarrhea monitoring. Legend: vaccination and blood collection from 50 to 60 days before calving, blood collection for IgG1 measurement against BCoV and BoRVA and monitoring of diarrhea

The study design is summarized in the Fig. 2. The study was carried out in a herd of beef cattle with Nelore animals, located in the city of Pirassununga-SP, Brazil (Latitude 21°59′46″ S, Longitude 47°25′36″ W). The use of animals was authorized by the owner. This herd was selected because the absence of previous historic of vaccination against NCD. The cows and heifers were submitted to the fixed-time artificial insemination (FTAI) protocols during the 2018 breeding season, with births scheduled from August up to October of 2019. Cows and heifers were managed extensively in separate paddocks formed by *Brachiaria decumbens*, receiving supplementation based on mineral salt, besides of hay in the dry periods. Initially a total of 126 cows and heifers were screened randomly to compose the experimental groups vaccinated (VAC) and non-vaccinated (NVAC). The animal’s vaccination was performed with a single dose of vaccine between 60 to 50 days before the expected calving, according to manufacturer’s instructions. Two mL of the vaccine was administered intramuscularly, on the left side of the neck in the VAC group. The vaccine is composed by the following formulation: inactivated BoRVA genotype G6P [5], inactivated Mebus strain from BCoV, ETEC K99 adhesin F5, and aluminium hydroxide and MontanideTM ISA70 VG as adjuvants. The NVAC group received 2 mL of saline solution 0.9%, administered as the same protocol from VAC group. In the final of the practical activities period, 117 calves were born to the cows and heifers screened initially and the groups were composed by 81 cows and 36 heifers distributed into vaccinated (VAC; cows = 40; heifers = 19) and non-vaccinated (NVAC; cows = 41; heifers = 17).

### Sampling from cows and heifers

Cows and heifer’s blood samples were collected from both experimental groups to determine the specific antibody titers (IgG1) against BoRVA and BCoV on the day of vaccination and 30 days after. The blood samples were collected by puncture of the external jugular vein in 10 mL tubes without anticoagulant using the vacuum collection system, after local antisepsis with 70% alcohol. Serum was obtained by using the centrifugation of samples at 2000x g for 10 minutes, and were stored in four 1.5 mL microtubes at − 20 °C.

### Calving monitoring, management and blood sampling

After vaccination, pregnant cows and heifers remained in separated prepartum paddocks, where they were monitored once a day in the morning to detect the births that occurred in the previous 24 hours. Dams and calves were transfer and maintained in the maternity paddocks in the first 48 to 72 hours after birth. During the ‘calf care’ (48 to 72 hours after birth), the calf blood samples were taken, and the passive immune transfer was evaluated. The calf care includes also navel disinfection using 10% iodine solution, weighing, ear tattoo (identification) and finally application of 1 mL Doramectin 1%. As inclusion criteria, the calves were clinically assessed, and only healthy animals were included. Clinical assessment included general condition, vital functions, hydration, mucous membranes, lymph nodes, and physical examination of the umbilical region. The blood samples were collected aseptically by puncture of the external jugular vein in 10 mL tubes without anticoagulant using the vacuum collection system. After sample collection, dams and calves were transferred to different postpartum paddocks, depending on the maternal categories (primiparous or multiparous). Despite of that, the nutritional management of the dams and calves was the same based on pasture and mineral supplementation.

### Passive immunity transfer evaluation

Blood samples were collected from calves in D2-D3. Serum samples were obtained by centrifugation at 2000x g for 10 minutes. Total serum protein (TSP) was estimated by using an optical refractometer scale from 0 up to 12 g/dL (RTP-12, Instrutherm). The remaining of serum samples was distributed in four microtubes 1.5 mL, and storage at −20oC for the titration of IgG1 against BCoV and BoRVA.

### Titration of specific antibodies

The IgG1 antibody (Ab) titers to BoRVA and BCoV were measured using a double sandwich ELISA [38–40].

### IgG1 to BoRVA Ab ELISA

IgG1 Ab titers to BoRVA were measured using a double sandwich ELISA. Briefly, 96-well Maxisorp NUNC plates (Thermo Scientific®, USA) were coated with 100 μL of guinea pig hyperimmune serum to BoRVA (1:5000 dilution in carbonate/bicarbonate buffer, pH 9.8) and incubated overnight (ON) at 4 °C. Plates were then washed and blocked with 10% non-fat milk solution diluted in PBS-0.05% Tween 20. After incubation during 1 hour at 37 °C, BoRVA Mebus strain-infected (107 FFU/mL) and mock-infected HRT-18 supernatants were added as positive and negative antigens, respectively. The virus was inoculated in the rows A, C, E and G; while negative controls (Mock) was added in the following rows B, D, F and H, incubated for 1 hour at 37 °C. The dilution used from both virus and mock was 1:4. After that, the plates were washed and the serum samples from cows and calves were added in duplicates (virus and mock) in the wells, starting at the titer from 4096 up to 262,144 (Log4), incubated for 1 hour at 37 °C. The HRP-conjugated commercial antibodies against bovine IgG1 diluted 1:3000 (Bethyl Laboratories, Inc., USA) were added and incubated for 1 hour at 37 °C. The reaction was developed using hydrogen peroxide and ABTS as a substrate/chromogenic system (Sigma Aldrich, USA), and the final reactions were read at a wavelength of 405 nm (Multiskan Ex, Labsystems Inc.). Positive and negative controls were also added in the plates. As a positive control, a serum from an animal presenting high titer of specific antibodies against BoRVA diluted 1:1024 was used. As negative control was used wash buffer solution. The titer of each sample was expressed as the 11% of the optical density (DO) from the positive control.

### IgG1 to BCoV

IgG1 Ab titers to BCoV were measured using a double sandwich ELISA. Briefly, 96-well Maxisorp NUNC plates (Thermo Scientific®, USA) were coated with 100 μL of specific IgY from hyperimmune egg yolk to BCoV (1:5000 dilution in carbonate/bicarbonate buffer, pH 9.8) and incubated overnight (ON) at 4 °C. Plates were then washed and blocked with 10% non-fat milk solution diluted in PBS-0.05% Tween 20. After incubation during 1 hour at 37 °C, BCoV Mebus strain-infected (107 FFU/mL) and mock-infected HRT-18 supernatants were added as positive and negative antigens, respectively. The virus were inoculated in the rows A, C, E and G; while negative controls (Mock) was added in the following rows B, D, F and H, incubated for 1 hour at 37 °C. The dilution used from both virus and mock was 1:2. Plates were washed and the serum samples from dams and calves were added in duplicates (virus and mock), and then the serial dilution were realized from titer 4096 to 262,144 (Log4), incubated for 1 h at 37 °C. The HRP-conjugated commercial antibodies against bovine IgG1 (Bethyl Laboratories, Inc., USA) diluted at 1:3000 were added and the plate were incubated for 1 hour at 37 °C. The reaction were developed using hydrogen peroxide and ABTS as a substrate/chromogenic system (Sigma Aldrich, USA), and the final reactions were read at a wavelength of 405 nm (Multiskan Ex, Labsystems Inc.). As a positive control were used serum from an animal with a high titer against BCoV (1:4096), and wash buffer solution as a negative control. The titer of each sample was expressed as the 11% of the optical density (DO) from the positive control.

### NCD monitoring and fecal sampling

NCD was monitored daily during the first 30 days of the calves’ life. When diarrhea was suspected, calves were captured for fecal evaluation by using Calf Heath Scoring Criteria. The score was classified from 0 to 3, according to fecal consistency, as follows: 0-normal consistency; 1-pasty and semi-formed consistency; 2-aqueous consistency with greater amount of water, in addition to fecal content adhered to the perineum and tail; and 3-fluid, fecal content adhered to the perineum and tail. Stool scores 0 or 1 were considered normal, while animals with scores of 2 or 3 were considered as calves with diarrhea [41]. Fecal samples were collected for the identification of diarrhea pathogens. The collection of fecal samples was made following sterile procedures. Initially, perineum area was cleaned followed by disinfection with chlorhexidine degermante 2% and alcohol 70%. Next, fecal samples were collected from the rectum using a sterile swab. The swap was transferred into an ampoule with Stuart medium for transportation and storage for the isolation of the *Escherichia coli.* In addition, a major amount of feces was obtained manually from rectus and stored in sterile universal collectors. In the laboratory, the feces from universal collectors were distributed into ten 2 mL microtubes and stored at -20 °C for subsequent diagnosis of BoRVA and BCoV. The remaining feces were conserved by the addition of dichromate 0.5% for the *Cryptosporidium* spp. diagnosis.

### Isolation of *E. coli*, DNA extraction and PCR for detection of virulence factor K99

The fecal swabs were streaked on MacConkey medium agar to isolated *E. coli* colonies, incubated for 24 at 37 °C. After, the lactose positive bacterial colonies were selected to perform biochemical tests such as Triple sugar iron (TSI), phenylalanine, citrate, motility, indole, lysine, ornithine and methyl-red media, incubated for 24 hours at 37 °C. The results were interpreted and the colonies identified as *E. coli* were inoculated in BHI medium with 15% glycerol, incubated for 24 hours at 37 °C. Finally, the strains were stored in a freezer at − 80 °C until the PCR procedures [42].

The DNA was extracted by using boiling method, and quantified using the QuBit dsDNA HS Assay Kit and the QuBit 2.0 fluorometer, following the manufacturer’s instruction (Life technologies). PCR was performed in a 25 μL reaction volume, containing 15 mM MgCl2 (2.5 μL), 200 μM dNTP (2.5 μL), 100 ng DNA template (1 μL), 0.5 U DNA polymerase Taq (0.25 μL) and 20 pmol (1 μL) of each primer for gene F5 (Forward – TATTATCTTAGGTGGTATGG; Reverse – GGTATCCTTTAGCAGCAGTATTTC). All samples and control were subjected to PCR cycles as follow: 94 °C for 5 minutes (denaturation), followed by 25 cycles of 94 °C for 30 seconds, 53 °C for 45 seconds, 70 °C for 1 minute (annealing) and 72 °C for 5 minutes (extension). The amplified products were visualized in 1.5% agarose gel, stained with Loading Dye (Synapse Biotechnology) and GelRedTM. The agarose gel was photographed by the Kodak Scientific System, and a 100 bp DNA ladder was used as a molecular size marker (100–1,000 bp).

### PCR for detection of BoRVA and BCoV and analysis from BoRVA VP6 gene

A multiplex semi-nested RT-PCR was performed for detection of BoRVA and BCoV [43]. Fecal samples were first prepared as 50% (liquid stool) or 20% (pasty stool) suspensions in DEPC-water (Diethylpyrocarbonate) and clarified at 5000x g/15 min at 4 °C, taking the supernatant as a sample. Total RNA extraction from the fecal sample suspensions was performed with TRIzolReagentTM (Invitrogen, Carlsbad/CA, USA), according to the manufacturer’s instructions. Kakegawa [44] and 8209 [45] strains were used as positive controls BCoV and BoRVA, respectively, and water-DEPC as negative control.

For the detection of BCoV, a set of three primers directed to the BCoV N-nucleocapsid protein-coding gene was used. The RT-PCR reactions for BoRVA detection were performed using three primers directed to the viral protein-coding gene VP1 (Table 4). Each RNA-containing tube was brought to 95 °C for 5 minutes for RNA denaturation, then immersed in ice and added from the 1x FirstStrand Buffer®, 1 mM of each dNTP, 10 mM reverse transcription combination reagent DTT, 1 μM of each primer (BCOV1 + BCOV2 for BCoV, ROT1 + ROT2 for rotavirus) and 400 U M-MLV Reverse Transcriptase® (Invitrogen®) for a final volume of 40 μL, reverse transcribed at 42 °C/60 minutes.

**Table 4:** Primers used for the detection of bovine coronavirus N-nucleocapsid, primers used for the detection of rotavirus VP1 viral protein-coding gene group A, fusion temperatures (Tm) and their amplicons (in base pairs)

Virus	Primer		Sequence 5′-3’	Location	T° melting	Amplicons
BCoV	BCOV1 (Sense)		AAGAGCTCAAYCCAAGCAAATGY	123–146	60 °C	463 pb
	BCOV2 (Antisense)		AGCAGACCTTCCTGAGCCTTCAAT	562–585	60 °C	463 pb
	BCOV3(Antisense)		TCAATRTCGGTGCCATACTGGTCT	405–428	59,9 °C	306pb (withBCoV1)
BoRVA	ROT1 (Sense)		CTCTGGCAAARCTGGTGTCA	737–753	59,7 °C	492 PB
	ROT2 (Antisense)		CATTCGACGCTGATGACATY	1206–1225	59,7 °C	492 PB
	ROT3(Antisense)		ARCAATCRACCAACCASTCCTGTA	938–961	59,8 °C	228pb (with ROT1)

After obtaining the complementary DNA, the PCR reaction was performed in a thermal cycler. To this end, 2.5 μL of the respective cDNA was added to the PCR reagent combination containing 1x PCR Buffer® (Invitrogen®), 0.2 mM from each dNTP, 0.25 μM from each pair of primers (BCOV1 + BCOV2 and BCOV1 + BCOV3 for BCoV, ROT1 + ROT2 and ROT1 + ROT3 for), 1.5mMMgCl2 and 0.5 U Platinum Taq DNA Polymerase® (Invitrogen®) to a final volume of 25 μL supplemented with DEPC-water.

The tubes were then brought to the thermal cycler for initial denaturation at 94 °C/4 min, followed by 35 cycles of 94 °C/30 sec (denaturation), 55 °C/30 sec with gradient 5 °C (hybridization) and 72 °C/45 sec (polymerization), followed by the cycle, 72 °C/5 min for the final extension. After electrophoresis on 1.5%, agarose gel stained with 0.5 μg/mL ethidium bromide and observed under ultraviolet light. The hemi-nested PCR for BCoV and rotavirus was performed by adding 2.5 μL of the first amplification product to the PCR reagent combination containing 1x PCR Buffer® (Invitrogen®), 0.2 mM from each dNTP, 0.25 μM of each primer (BCOV1 + BCOV3 for BCoV, ROT1 + ROT3 for rotavirus), 1.5mMMgCl2 and 0.5 U Platinum Taq DNA Polymerase® (Invitrogen®) and sterile ultrapure water (to a final volume of 25 μL), leading to the thermal cycler initial denaturation of 94 °C/4 min followed by 25 cycles of 94 °C/30 sec (denaturation), 55 °C/30 sec with gradient of 5 °C (hybridization) and 72 °C/45 sec (polymerization) followed by 72 °C/5 min for the final extension. After electrophoresis on 1.5%, agarose gel stained with 0.5 μg/mL ethidium bromide and observed under ultraviolet light. For analysis of the BoRVA VP6 gene, three positive samples were subjected to genetic sequencing [46] to verify the nucleotide identity between the field viral strain and the vaccine strain.

### Detection of *Cryptosporidium* spp.

The detection of *Cryptosporidium* spp. was performed by the sucrose saturated flotation technique [47]. To do so, initially, the feces were washed to remove the fat and facilitate the observation of protozoa. One to two grams of feces were added to a collecting beaker with 8 to 9 mL of distilled water and with the aid of a glass stick, the feces were utterly diluted. The solution was filtered, and the liquid fraction added into a 15 mL plastic tube, then 4 mL of ether was added to the tube and then homogenized. Samples were centrifuged for 5 min at 796 x g. After this process, the fat layer and the supernatant were removed with the aid of a Pasteur pipette, and the walls of the plastic tube were wiped with gauze. The stool “pellet” was added in 9 mL saturated sucrose solution, homogenized and centrifuged for 10 min at 264 x g. The supernatant was removed with a metal handle and placed on a glass slide for observation under a 400 x magnification optical microscope.

### Statistical analysis

Statistical analyses of qualitative nominal data were performed using the SPSS program (IBM Corp. Released 2011. IBM SPSS Statistics for Windows, Version 19.0. Armonk, NY: IBM Corp.). The association between experimental groups (NVAC and VAC) with diarrhea incidence and failure of passive immune transfer using four different cut-off points (5.5, 6.0, 6.5, 7.0 g/dL) were evaluated by using Chi-square test or Fisher’s exact test, after transforming of the variables into a dichotomous variable (negative or positive for failure of passive immune transfer). The Fisher’s exact test was chosen when the groups had less than 5 animals. Frequencies of etiological agents of diarrhoea between groups (VAC and NVAC) also was compared using the same statistical tests.

To evaluate the serological response induced by pre-partum vaccination, data from dams was distributed in three Excel files according to maternal categories (heifers, cows or both), which was evaluated in separated statistical analysis.

Statistical analysis of continuous data was performed using SAS version 9.4 for Windows (SAS® version 9.4, SAS Institute Inc., Cary, NC, USA) with models fitting a Gaussian distribution. Data were tested for normality of residuals using the GLM procedure. Homogeneity of variances followed Hovtest and Welsh methods, and normality of residuals was analysed using the PROC UNIVARIATE procedure of SAS following the Shapiro-Wilk method. Specific antibodies titers against BoRVA and BCoV had non-parametric profile and were submitted to a logarithmic transformation by log10 to obtain normal distribution. These variables were tested for fixed effects of treatments (VAC and NVAC) and times in the pre-partum period (d-60 and d-30), as well the interaction between groups and time (Groups x Times) using the MIXED procedure. Covariances matrices were tested and defined according to the lowest value obtained for “Akaike’s Information Criterion (AIC)”. When the interaction between maternal categories and times were detected, the difference between groups in each time of evaluation was detected by using Student T test in each prepartum moment. Student T test was also used to perform the comparison between continuous data from calves at 48 hours of life. All statistical differences were considered significant when *P* ≤ 0.05.

## Supplementary Information

**Additional file 1.**

**Additional file 2.** 

## Data Availability

The database used for this study is available in the supplementary material (Banco de dados 21.07.2021.xls).

## Abbreviations

NCD: Neonatal Calf Diarrhea
BoRVA: Bovine Rotavirus type A
BCoV: Bovine Coronavirus
VAC: Vaccinated group
NVAC: Non-vaccinated group
W/O: Water-in-oil
Ab: Antibody
ELISA: Enzyme Linked Immuno Sorbent Assay
TSP: Total serum protein
FPIT: Failure of the passive immune transfer
AI: Artificial insemination
60PP: 60 days before expected calving
30PP: 30 days before expected calving
