# Supplementary material for: Efficacy of prepartum vaccination against neonatal calf diarrhea in Nelore dams as a prevention measure
Source: BMC Vet Res. 2022 Aug 22;18:323. doi: 10.1186/s12917-022-03391-5 (PMC9394007; doi:10.1186/s12917-022-03391-5)
Supplement: Supplementary file 1 — Additional file 1. [file 12917_2022_3391_MOESM1_ESM.docx]

SUPLEMENTARY MATERIAL

Table 1 - Optimal sample size considering a power of test of 80%.

| Category | E. Group | Diarrhea Incidence | Optimal Sample Size | Test Power |
| --- | --- | --- | --- | --- |
| Heifers | VAC | 26.30 | 157 | 0.80 |
|  | NVAC | 41.20 | 157 |  |
| Cows | VAC | 15.00 | 303 |  |
|  | NVAC | 24.40 | 303 |  |
| Total | VAC | 18.60 | 249 |  |
|  | NVAC | 29.30 | 249 |  |

Notes: The calculation was performed in the R-3.6.3 software using the following syntax: power.prop.test (power=0,8, p1=x, p2=y).

Table 2 - Mean (±STD Err) of specific antibodies titers against Coronavirus and Rotavirus (Log10) in Nelore dams non-vaccinated (NVAC) or vaccinated (VAC) in the pre-partum period.

| Dams Categories | Variables | Times | NVAC | VAC | P-value* |
| --- | --- | --- | --- | --- | --- |
| Heifers | BCoV | 60PP | 4.6040±0.1360 | 4.3729±0.1013 | 0.1762 |
|  | BCoV | 30PP | 4.4977±0.1168 | 4.6897±0.0985 | 0.2146 |
|  | BoRVA | 60PP | 4.1790±0.1313 | 3.8975±0.1250 | 0.1299 |
|  | BoRVA | 30PP | 4.1436±0.1017 | 4.2778±0.1209 | 0.4076 |
| Cows | BCoV | 60PP | 4.5375± 0.0761 | 4.3800± 0.0681 | 0.1276 |
|  | BCoV | 30PP | 4.1704± 0.0609 | 4.7713± 0.0899 | <0.0001 |
|  | BoRVA | 60PP | 4.3759± 0.0516 | 4.2445± 0.0745 | 0.1516 |
|  | BoRVA | 30PP | 4.2732± 0.0461 | 4.6509± 0.0777 | <0.0001 |
| Total Dams | BCoV | 60PP | 4.5570± 0.0664 | 4.3777± 0.0561 | 0.0411 |
|  | BCoV | 30PP | 4.2663± 0.0579 | 4.7450± 0.0684 | <0.0001 |
|  | BoRVA | 60PP | 4.3182± 0.0537 | 4.1328± 0.0674 | 0.0339 |
|  | BoRVA | 30PP | 4.2352± 0.0443 | 4.5308± 0.0688 | 0.0005 |

*Student T Test; 60PP – 60 days before expected calving; 30PP – 30 days before expected calving.

Table 3 - Vital function (Mean ± SDT Err) of neonatal Nelore calves from non-vaccinated (NVAC) and vaccinated (VAC) dams in the pre-partum period against neonatal diarrhea.

| Parameters | Dams categories | NVAC | VAC | P-value | Reference* |
| --- | --- | --- | --- | --- | --- |
| FC | Heifers | 165.9±7.3254 | 137.9±5.4493 | 0.0038 | 116.72 ± 17.68 |
|  | Cows | 144.1± 4.2770 | 144.9± 4.5954 | 0.8985 |  |
|  | Total | 150.5± 3.9006 | 142.6± 3.5748 | 0.1409 |  |
| FR | Heifers | 39.5294± 3.1226 | 46.7368± 3.1501 | 0.1147 | 38.33±10.39 |
|  | Cows | 46.3415± 2.0952 | 44.8000± 1.7611 | 0.5758 |  |
|  | Total | 44.3448± 1.7744 | 45.4237± 1.5556 | 0.6480 |  |
| T°C | Heifers | 38.8412±0.0809 | 38.9579±0.1176 | 0.4298 | 39.47±0.22 |
|  | Cows | 38.9805± 0.0538 | 38.9000± 0.0648 | 0.3413 |  |
|  | Total | 38.9397± 0.0452 | 38.9186± 0.0575 | 0.7750 |  |

*T Student Test; ** Gasparelli et al. (2009)

Table 4 - Mean and STD Error for the specific antibodies titers (Log 10) in Nelore calves born from vaccinated (VAC) and non-vaccinated (NVAC) dams in the pre-partum period.

| Dams Categories | Groups | Specific antibodies against vaccine antigens (Log10) | |
| --- | --- | --- | --- |
|  |  | Coronavirus | Rotavirus |
| Heifers | NVAC | 4.7456±0.1354 | 4.3207±0.0772 |
|  | VAC | 4.7531±0.1118 | 4.3095±0.1401 |
|  | P-value* | 0.9661 | 0.9448 |
| Cows | NVAC | 4.4053± 0.0973 | 4.3613± 0.0460 |
|  | VAC | 4.7713± 0.0973 | 4.6660± 0.0799 |
|  | P-value* | 0.0095 | 0.0016 |
| Total | NVAC | 4.5051± 0.0814 | 4.3494± 0.0393 |
|  | VAC | 4.7655± 0.0746 | 4.5512± 0.0732 |
|  | P-value* | 0.0200 | 0.0171 |

*T Student Test
